# Investigating Combined Impact of Adverse Road-Weather Conditions and Heavy Vehicles on Saturation Headway

**DOI:** 10.1177/03611981221089303

**Published:** 2022-05-18

**Authors:** Ryutaro Hirose, Babak Mehran, Agnivesh Pani

**Affiliations:** 1 Department of Civil Engineering, University of Manitoba, Winnipeg, MB, Canada; 2 Department of Civil Engineering, Indian Institute of Technology (IIT) BHU, Varanasi, India

**Keywords:** operations, arterials, signalized, capacity, intersections, level of service

## Abstract

Adverse road-weather (RW) conditions make driving behavior more conservative and the headway during saturated conditions longer, leading to a significant reduction in the capacity of signalized intersections. Past studies indicate that the degree of the influence of adverse RW conditions on intersection performance changes by heavy vehicle (HV) ratio in traffic flow. However, little is known about the combined impacts of adverse RW conditions and HV ratio on saturation headway and how they can be considered in the planning of signalized intersections in areas with long winter. To fill this research gap, in this study the saturation headway data for over 2,000 signal cycles were extracted from video recordings at two signalized intersections in Winnipeg, Canada. The combined impacts of adverse RW conditions and HV ratios are statistically investigated in the paper with due focus given to saturation headway distributions and models. To account for differences in vehicle type, passenger car equivalent and headway distributions are evaluated under different RW conditions. The analysis findings suggest that the saturation headways increased by up to 38.7% as a result of adverse RW conditions. The multiple regression analyses incorporating HV ratios quantify the relationship between saturation headway and various sets of explanatory variables covering adverse RW conditions and roadway geometric factors. The model estimation results reveal that HVs are less sensitive to RW conditions than passenger vehicles. Overall, the study findings will help in designing signalized intersections under adverse RW conditions with various HV ratios.

In the planning and design of signalized intersections, saturation flow rate (SFR), which is defined as “the rate at which vehicles that have been waiting in a queue during the red interval cross the stop line of a signalized intersection approach lane during the green interval,” is essential for calculating intersection capacity (*1*). SFR for a specific lane (or lane group) is estimated considering base SFR (vehicles per hour), that is, the inverse of saturation headway, intersection geometry (e.g., lane width and grade), traffic flow characteristics (e.g., right/left-turn volume and traffic composition), and lane utilization pattern. The calculation methods for SFR in most intersection planning manuals are based on traffic analysis results under the assumption of ideal road-weather (RW) conditions. The application of this approach to winter weather conditions has been questioned, since it is hypothesized that SFR varies greatly depending on road surface and weather conditions (*1*). This is a significant concern for North American regions with extreme weather conditions in winter such as most northern U.S. states and the Canadian provinces. For example, winter season with extreme cold and snowy conditions can last for about half a year in the Candian prairie provinces (i.e., Manitoba, Saskatchewan, and Alberta). It is well documented that poor visibility resulting from snowfall, and icy roads have negative impact on traffic operations and safety (*2*, *3*). Thus, the importance of considering RW conditions in signal timing design and operational planning of signalized intersections is well-recognized. The Canadian Capacity Guide for Signalized Intersections advises determining applicable SFR values depending on RW conditions to assess potential difficulties, which rarely arise under typical dry weather conditions (*1*). On the other hand, it is assumed that the degree of the influence of RW conditions on SFR changes by heavy vehicle (HV) ratio in traffic flow and differs between passenger cars (PCs) and HVs. However, the impacts of HVs on saturation headways observed at signalized intersections under adverse RW conditions are still not fully quantified in the academic literature. Furthermore, passenger car equivalent (PCE) for HVs, which is the number of PCs having equivalent impact on the traffic stream as an HV, is generally adopted as a constant value of 2.0 regardless of RW conditions (*4*).

To address this discernible research gap, this study analyzes saturation headway variations under adverse RW conditions and different HV ratios using video recordings at two signalized intersections in Winnipeg, Manitoba. Observed saturation headways are classified based on RW conditions and vehicle type. Derived PCE values under different adverse RW conditions are compared with Highway Capacity Manual (HCM) values, and the relationship between saturation headway and HV ratios is examined under different RW conditions (*4*). Finally, different saturation headway models are developed to quantify the compound impact of RW conditions and HV proportions. The study findings are expected to provide a better insight into factors influencing the capacity of signalized intersections in cold regions in North America and elsewhere in the world with comparable RW conditions, especially where the impacts of adverse RW conditions and HV traffic are combined.

The remainder of the paper is structured as follows: first, an overview of the study background is provided with key focus on literature related to the impact of adverse weather conditions on traffic parameters. The study methods, including data collection, are presented in the subsequent section, followed by discussions on implications of RW conditions and HV traffic, and their varying degree of influence. The section after that describes saturation headway distributions analysis and modeling, and the last section concludes the paper.

## Literature Review

Several studies have investigated the implications of adverse RW conditions on intersection operations including the impacts on traffic parameters such as SFRs, start-up delay, and free-flow speed. Perrin et al. estimated the reduction in SFR ranged from 6% (rain) to 20% (snowy and sticking), speed was reduced by 30% at most, and start-up lost time increased by 23%, in Salt Lake City, Utah (*5*). In an analysis in Waterloo, Ontario, Lu et al. quantified weather impacts on signal-design-related traffic parameters analyzing video recordings of a signalized intersection (*6*). They found SFR decreased by 17% on slushy roads and 25% on the snowy road compared with ideal RW conditions. Further, in their study, they observed that free-flow speed declined as much as 23% on the snowy road, while start-up delay showed little change. In New England, Agbolosu-Amison et al. also confirmed that inclement RW conditions caused a reduction in saturation headways in the range of 2%–21% combined with rain intensity (*7*). Chodur et al. focused more on comprehensive analysis of SFR in Polish intersections (*8*). In their research, it was found that adverse weather conditions produced a shorter initial interval where start-up lost time appeared and a shorter middle interval, that is, the interval during which saturation headway is stable in a cycle. Prevedouros and Chang compared wet road conditions with the usual dry condition and reported 4.7% reduction in the capacity of arterial streets in Honolulu, U.S. (*9*). Their linear regression headway model suggested that observed headways are 4% longer in rainy or wet conditions. Further, they confirmed that wet conditions deteriorated the level of service by one level in four out of five intersections surveyed in their study (*9*). For HV implications under different RW conditions, Asamer and Van Zuylen investigated 2 h of video recording with 100 signal cycles in Vienna, Austria, and measured PCU (Passenger Car Unit) values under dry, wet, and snowy conditions (*10*). However, they concluded that the larger PCU values with higher standard deviations observed under dry conditions resulted from lacking sufficient sample size. In another research effort in Asia, Alhassan and Ben-Edigbe found that PCE values decreased with an increase in rainfall intensity in a highway section in Malaysia (*11*).

In addition, the need for weather-responsive trassic signal management was highlighted in past research (*12*). Lu et al. implemented an adjusted signal timing in response to weather-related delays in Synchro and VISSIM traffic microsimulation software, and measured intersection delay in a simulation environment for two scenarios, that is, an isolated signalized intersection and a coordinated corridor with four signalized intersections (*6*). They showed that their proposed weather-adjusted signal timing plan could reduce the delay by up to 19.2% when traffic demand was relatively high or medium in both scenarios. In addition to reduced delay, Agbolosu-Amison et al. found that their proposed weather-adjusted signal timing plan could reduce the number of stops and fuel consumption by up to 8.3% and 4.5%, respectively (*7*).

Review of existing studies confirms the need for further research to analyze the impact of weather-related variations in traffic performance and operation of signalized intersections in winter. There are limited studies focusing on the impact of snow and ice, as most existing studies only consider rain and wet road surface conditions. In addition, the combined impact of RW conditions and traffic composition—that is, HV ratio—has not been studied adequately. Yet, there is concensus that HVs noticeably influence traffic parameters as a result of their larger vehicle size and limited maneouverability (*4*, *11*).

## Data and Methods

### Location and Overview of the Study Intersections

**Figure 1.**
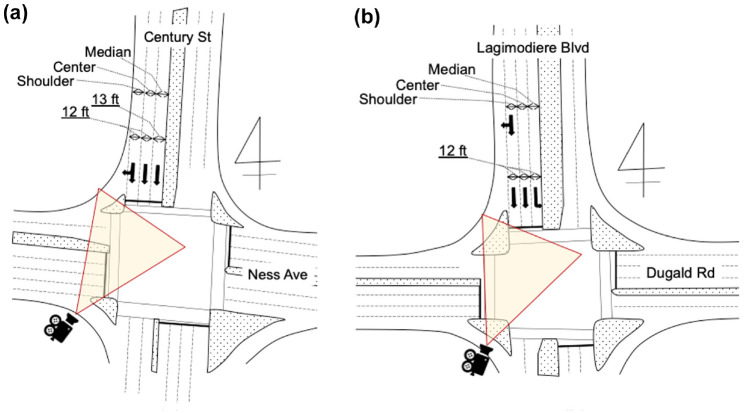
Sketches of intersections: (*a*) Century and (*b*) Dugald.

**Figure 2.**
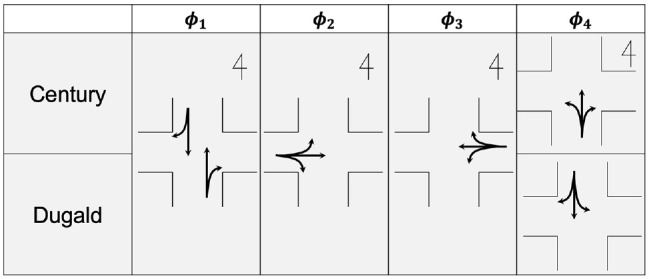
Phase diagrams for signal operations at Century and Dugald.

This analysis focuses on two signalized intersections in Winnipeg, Canada: Century St and Ness Ave (Century), and Dugald Rd and Lagimodiere Blvd (Dugald) as shown in Figure 1. The target intersections have a lane width of 12 ft or 13 ft, and the southbound approaches are assigned for exclusive through, shared through with right turn, and exclusive left-turn traffic. Traffic signal operation at both intersections is similar, as shown in the phase diagrams in Figure 2. At the beginning of the green phase, the traffic stream was primarily saturated with queues during most of the observation periods. Both intersections carry significant volumes of PCs and HVs. In particular, on the southbound center lane next to the shoulder lane at Century, 26% of all observed cycles contained at least one HV.

### Data Collection for Saturation Headway

Headway data was extracted from video recordings captured by overhead cameras fixed ahead of the direction of flows at the target intersections. A total of 71 h of video footage was taken in the morning (07:30–09:30) and afternoon (15:30–17:30) on multiple days in March 2019 and January 2020. The data were collected in January and March to ensure capturing various RW conditions required for the analyses in this study. The observations are made by continuously measuring the headway of queueing vehicles passing through the intersection for each lane. TrafficAnalyzer—a video processing software developed by Suzuki and Nakamura—was used to process the recordings (*13*). In this study, the saturated headway is calculated based on the following procedure recommended in the HCM (Highway Capacity Manual) (2016).

1. Manually record the timestamps when the front axle of each vehicle in the queue crosses the stop line at the beginning of the green phase.
2. For each vehicle discharged from the queue, the headway is estimated as the difference between its timestamp and that of its leading vehicle recorded in Step 1.
3. The average headway of the fifth and subsequent queuing vehicles in each cycle is estimated as satutarion headways for that cycle as per HCM (2016) definitions. In this study, only queues with eight or more vehicles were considered, to ensure the validity of the average headway in each cycle.

In addition to the headway data, three types of vehicle type were also registered based on the Federal Highway Administration (FHWA) classification: PCs, single-unit trucks (STs), and articulated trailers (ATs) (*14*). From a sample size standpoint, STs and ATs are combined as HVs in this study.

### Data Collection for Weather Conditions

RW conditions were classified into seven categories based on the observations from videographic data and the criteria described below:

- Dry = no moisture, ice, or snow are detected on the road surface.
- Partly wet = moisture is detected in the wheel path.
- Wet = the entire road surface is wet.
- Icy = the road surface is flat, but the road surface is white and frozen.
- Partially snow-covered = the road surface is elevated with dirt snow, and the wheel path is melted.
- Packed snow = more than 80% of the road surface is covered with white snow.
- Snow-covered = the road surface is completely covered with white snow.

**Figure 3.**
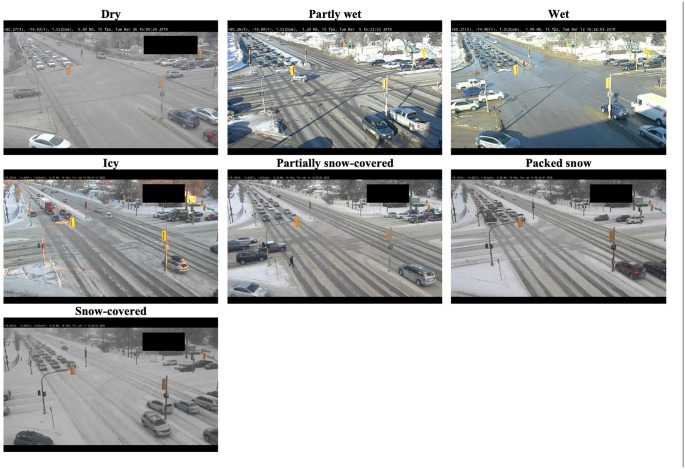
Screenshots of road-weather (RW) classifications at Century.

Field surveys were conducted on only selected days in March 2019 to ensure the consistency of RW classifications. RW condition categories are presented visually in Figure 3.

To supplement the classification of RW conditions, essential weather information was also obtained from Environment Canada (*15*).

## Preliminary Analysis and Visualizations

### Impacts of Road Weather Conditions

**Figure 4.**
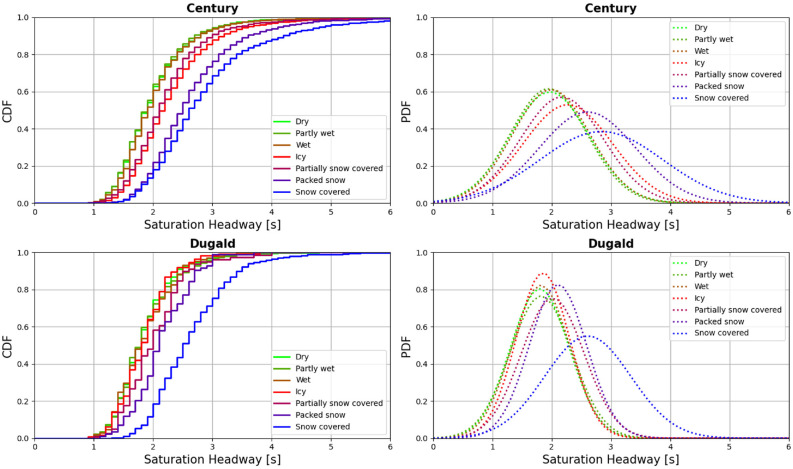
Cumulative density function (CDF) and probability density function (PDF) of saturation headway distributions at Century and Dugald.

Saturation headways measured per cycle were categorized into seven groups as per RW condition classification. In Figure 4, the stepped lines show the cumulative percentage of saturation headway frequency at 0.1 s increments. The dotted curves are the modeled probability density functions (PDFs) assuming a normal distribution for saturation headway. Table 1 shows the number of cycles, mean values, standard deviations of saturation headways, and the increase rate for each intersection and road surface condition. The increase rate can be calculated by dividing the saturation headway difference between dry and adverse weather conditions by that of dry conditions. Generally, observed saturation headways are relatively longer and with higher standard deviations under adverse RW conditions compared with dry conditions. The Dwass Steel all-pairs comparison test for non-parametric data was performed for saturation headways in different groups (*16*). The results indicated that, for Century, there are significant differences among the mean values of three groups (dry, partly wet, and wet), (icy, partially snow-covered), and (packed snow, snow-covered) at 95% confidence level. For Dugald, significant differences were barely detected between dry and partially snow-covered. This observation could be the result of smaller sample size of fewer than 20 cycles. Thus, additional categories were created to combine RW conditions, that is: a “normal” category to combine dry, partly wet, and wet; a “partly snowy” category to combine icy and partially snow-covered; and a “snowy” category to combine packed snow and snow-covered. At Century, the mean value of the saturation headways for the normal category was 2.02 s, while that of partly snow and snowy categories were increased by 13.6% and 38.4%, respectively. In the case of Dugald, the mean value of saturation headway increased by 17.6% and 38.7%, respectively, compared with 1.81 s under the normal category. Kolmogorov–Smirnov (KS) test results in Table 2, which uses the maximum absolute difference between the two cumulative distributions (KS stats = Kolmogorov-Smirnov statistics), confirms that the two groups of normal, and partly snowy or snowy were not derived from the same distribution for both intersections.

**Table 1.** Summary of Collected Data for the Center Lane

Road-weather (RW) classifications	Century (Figure 2*a*)					Dugald (Figure 2*b*)				
	Number of cycles	Mean headway (s)	SE	Mean headway (s)	Increase rate (%)	Number of cycles	Mean headway (s)	SE	Mean headway (s)	Increase rate (%)
Normal										
Dry	139	2.02	0.72	1.99	na	204	1.93	0.5	1.93	na
Partly wet	216	1.99	0.71			82	1.97	0.52		
Wet	155	2.04	0.7			16	2.15	0.49		
Partly snowy										
Icy	188	2.34	0.82	2.26	Δ13.6	16	2.29	0.53	2.27	Δ17.6
Partially snow covered	80	2.24	0.9			22	2.27	0.45		
Snowy										
Packed snow	74	2.68	0.9	2.75	Δ38.4	15	2.48	0.49	2.68	Δ38.7
Snow covered	108	2.81	1.01			35	2.69	0.73		

*Note*: SE = standard error; na = not applicable.

**Table 2.** Kolmogorov–Smirnov (KS) Test Results

	Place^a^				Lane types		Vehicle types^a^			
	Century		Dugald		Median versus center		Passenger car (PC)		Heavy vehicle (HV)	
	KS stats	P-value	KS stats	P-value	KS stats	P-value	KS stats	P-value	KS stats	P-value
Normal	na		na		0.177	0.000	na		na	
Partly snowy	0.571	0.000	0.330	0.001	0.192	0.000	0.218	0.000	0.110	0.778
Snowy	0.833	0.000	0.823	0.000	0.093	0.514	0.440	0.000	0.201	0.124

a The reference of KS results for place and vehicle types are Normal conditions. *Note*: na = not applicable, KS stats = Kolmogorov-Smirnov statistics.

### Degree of Influence of Road Surface Conditions

**Figure 5.**
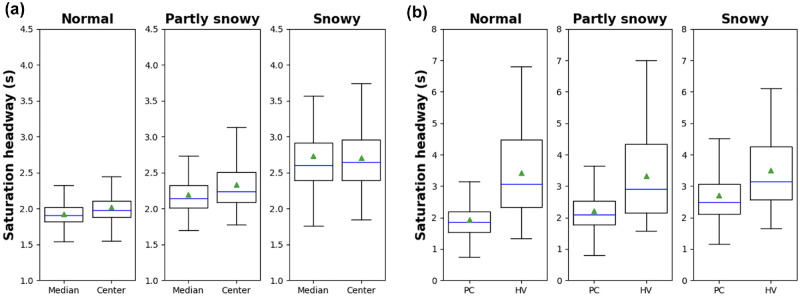
Boxplots of headways at Century: (*a*) lane type and (*b*) vehicle type.

In this section, the degree of influence of RW conditions on saturation headway is analyzed for each lane and vehicle type. Figure 5 represents boxplots of observed saturation headways at Century, where, in each box, the height of the box represents the interquartile range, the line dividing the box indicates the median, the triangle represents the mean, and the whiskers show the maximum and minimum values (excluding the outliers). Figure 5*a* shows the saturation headway per cycle for the through lanes, which are the median lane next to the median island, and the center lane next to the shoulder lane at Century. In normal and partly snowy conditions, there was a significant difference between median and center lanes according to the results of the KS test shown in Table 2, but no significant difference was observed in snowy conditions. This may be caused because the median lane is usually used as an overtaking lane. Thus, the median lane could potentially represent relatively aggressive driving behaviour. However, when snow accumulates on the road, the boundary between the lanes and the median island becomes blurred, and the median lane does not fully function as an overtaking lane.

The degree of influence of RW conditions on saturation headway was also investigated considering vehicle type. Figure 5*b* shows the headway distribution for PC-only and HV-only for each RW condition of the center lane at Century, which has sufficient HV samples. Measured headways were labeled as PC-only or HV-only based on the vehicle type observed. It can be seen that saturation headway increased considerably by the vehicle size. In Table 2, headway distributions of each vehicle type are compared using the KS test under adverse RW and normal condition. However, the impact of RW conditions was more pronounced for the PCs, while the observed difference for HVs decreased gradually. Besides, PCE values were calculated using Equation 1 from headways and HV ratio under three RW conditions.

(1) $$\begin{array}{c} h_{s}=\;h_{\mathrm{PC}}\left(1-P_{\mathrm{HV}}\right)+h_{\mathrm{PC}}P_{\mathrm{HV}}\cdot \mathrm{PCE}\; \end{array}$$

where

$h_{s}$ = average saturation headways per cycle,

$h_{\mathrm{PC}}$ = average headways of only PCs in the cycle, and

$P_{\mathrm{HV}}$ = the proportion of HVs in the cycle.

As shown in Table 3, the mean PCE for normal condition was 2.07, that is, consistent with the default value of 2.0 in HCM (2016) for the HV ratio adjustment. However, mean PCE in partly snowy and snowy conditions reduced to 1.72 and 1.47, respectively. The higher standard deviation still exists, but these results support the notion that HVs are less susceptible to adverse RW conditions. There is a possibility that the stability of HVs resulting from their weight and size, as well as their higher viewing angle, and the experience of HV drivers result in more stable driving behavior for HVs at intersections even under adverse RW conditions compared with PCs.

**Table 3.** Summary of Passenger Car Equivalent (PCE) and Linear Regression Models

	Cycles	PCE		(Saturation headways) = coef. (heavy vehicle [HV] ratio) + intercept			
		Mean	SE	Coef.	Intercept	R-squared	P-value
Normal	121	2.07	0.90	0.034	1.93	0.63	0.000
Partly snowy	74	1.72	0.81	0.019	2.23	0.46	0.000
Snowy	51	1.47	0.48	0.011	2.67	0.18	0.005

*Note*: SE = standard error; Coef. = coefficient.

**Figure 6.**
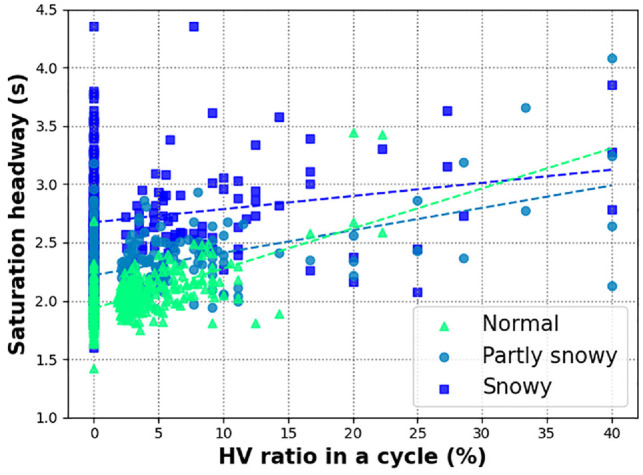
Saturation headway and heavy vehicle (HV) ratio per cycle at Century.

In addition, to see the overall trends between HV ratio and saturation headways for different RW conditions, linear regression models were developed using the least-squares method. The resulting models are shown in Figure 6 and the estimated parameters are listed in Table 3. Although the coefficient of determination for saturation headway under snowy conditions is low, the slope of regression lines demonstrates the impact of HV ratio on saturation headway under different RW conditions. Additionally, it can be observed that the impact of HV ratio on saturation headway is less significant as RW conditions aggravated, that is consistent with observations presented earlier. While the regression model coefficients are statistically significant in the headway model, caution is recommended while using these models for field prediction during partly snowy and snowy conditions, since the explanatory values (R-squared) of the models are limited.

## Results and Discussions

### Saturation Headway Distributions

**Figure 7.**
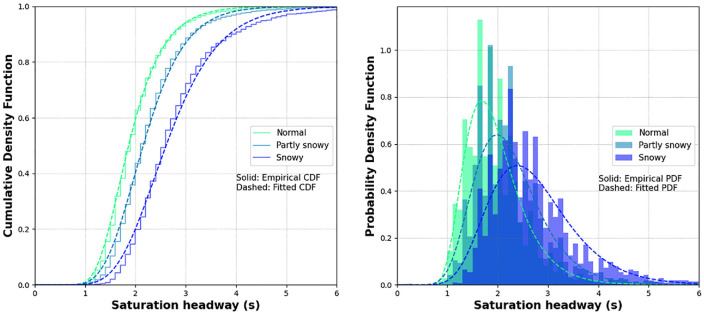
Density functions of the best fitted models (lognormal function).

In the first part of analysis, this paper develops different saturation headway distributions because headway generation is a first step to simulate traffic flow realistically. As shown in Table 4, several functional forms can be considered to model saturation headway distribution for through movements. In this study, five alternative models—normal, lognormal, gamma, logistic, and Weibull distribution functions—were examined, and model parameters were calculated using the maximum likelihood method. The goodness of fit for each distribution function was evaluated using the KS test. In the end, since the P-values of all the models were less than 0.05, the model with the smallest KS statistic was considered the best fitting model. Consequently, the lognormal distribution function defined by Equation 2 was found to be the best model for representing saturation headway distribution in all case. Figure 7 compares observed cumulative and probability distributions with fitted models using the lognormal distribution function for each RW condition under different HV ratios. It can be seen that, overall, saturation headway increases as RW conditions degrade and the probability functions become flat, but the difference in the distributions between non-HV-involved headway and HV-involved headway was negligible.

(2) $$\begin{array}{c} f\left(x\right)=\frac{1}{\sqrt{2\pi }\sigma x}\exp\left\{-\frac{{\left(\ln x-\mu \right)}^{2}}{2\sigma^{2}}\right\} \end{array}$$

(3) $$\begin{array}{c} \mu =\ln\left(\frac{m^{2}}{\sqrt{\nu +m^{2}}}\right),\sigma =\sqrt{\ln\left(\frac{\nu }{m^{2}}+1\right)} \end{array}$$

**Table 4.** Evaluation of the Fitting of Headway Distributions

	Distribution function	Normal			Partly snowy			Snowy		
		KS stats	P-value	Rank^a^	KS stats	P-value	Rank^a^	KS stats	P-value	Rank^a^
Non-HV-involved headway	Normal	0.0960	0.0000	5	0.0929	0.0000	4	0.1134	0.0000	4
	Lognormal	0.0344	0.0000	1	0.0412	0.0000	1	0.0591	0.0000	1
	Gamma	0.0501	0.0000	2	0.0541	0.0000	2	0.0747	0.0000	3
	Logistic	0.0605	0.0000	3	0.0569	0.0000	3	0.0736	0.0000	2
	Weibull	0.0865	0.0000	4	0.0978	0.0000	5	0.1162	0.0000	5
	Lognormal parameters	( μ,σ ) = (0.4452, 0.3283)			( μ,σ ) = (0.9162, 0.2462)			( μ,σ ) = (1.0734, 0.2718)		
HV-involved headway	Normal	0.1157	0.0000	5	0.1057	0.0000	4	0.1171	0.0000	4
	Lognormal	0.0382	0.0000	1	0.0472	0.0000	1	0.0620	0.0000	1
	Gamma	0.0619	0.0000	2	0.0634	0.0000	2	0.0780	0.0000	3
	Logistic	0.0689	0.0000	3	0.0639	0.0000	3	0.0764	0.0000	2
	Weibull	0.1081	0.0000	4	0.1153	0.0000	5	0.1174	0.0000	5
	Lognormal parameters	( μ,σ ) = (0.4185, 0.3554)			( μ,σ ) = (0.8703, 0.2706)			( μ,σ ) = (1.0707, 0.2797)		

*Note*: HV = heavy vehicle; KS stats = Kolmogorov–Smirnov statistics.

a Rank compares KS stats among the distribution functions with P-value of less than 0.10.

where

m = mean of headway distribution, and

ν = variance of headway distribution.

### Saturation Headway Models

In this second part of the analysis, saturation headways of the through-lane $h_{s}$ are modeled by multiple regression models using the least-squared method to asess the simultaneous impact of geometric design and traffic volume on saturation headway. After several iterations considering different explanatory variables including:

- HV ratio (%)
- Partly snowy dummy variable (not partly snowy = 0, partly snowy = 1)
- Snowy dummy variable (not snowy = 0, snowy = 1)
- Time dummy variable (PM = 0, AM = 1)
- Place dummy variable (Century = 0, Dugald = 1)
- Lane width (difference relative to 12 ft)
- Daytime dummy variable (daytime = 0, not = 1)
- Weekend dummy variable (weekend = 0, not = 1)
- Temperature (°C),

independent variables with better prediction power were selected using the stepwise method as shown in Equation 4, and a multiple regression analysis was conducted.

(4) $$\begin{array}{c} h_{s}=\alpha_{0}+\alpha_{1}x_{1}+\alpha_{2}x_{2}+\alpha_{3}x_{3}+\alpha_{4}x_{4}+\alpha_{5}x_{5}+\alpha_{6}x_{6} \end{array}$$

where

$\alpha_{0}\sim \alpha_{6}$ = model coefficient,

$x_{1}$ = HV ratio (%),

$x_{2}$ = partly snowy dummy variable,

$x_{3}$ = snowy dummy variable,

$x_{4}$ = time dummy variable,

$x_{5}$ = place dummy variable,

$x_{6}$ = lane width (ft).

The results of the following three models are shown in Table 5:

**Table 5.** Results of Multiple Linear Regression

Variables	Range or frequency	Sig^b^	Estimated parameters		SE	t-value	t-value ratio (%)
Model I (Except heavy vehicle [HV]-containing cycles)							
Intercept	na	na	$\alpha_{0}$	1.952	na	na	na
Partly snowy dummy	450 cycles	*	$\alpha_{2}$	0.327	0.016	20.631	22.6
Snowy dummy	291 cycles	*	$\alpha_{3}$	0.857	0.019	44.473	48.7
Time dummy (PM = 0, AM = 1)	173 cycles	*	$\alpha_{4}$	−0.350	0.024	−14.637	16.0
Place dummy (Century = 0, Dugald = 1)	368 cycles	*	$\alpha_{5}$	−0.156	0.018	−8.864	9.7
Lane width (ft)^c^	0–1 ft	*	$\alpha_{6}$	−0.039	0.014	−2.809	3.1
Number of observations (cycles): 1,898 Prob(F-stats): 0.000	Adjusted R-squared: 0.548 Mean absolute percentage error (MAPE): 8.24% Root mean squared percentage error (RMSPE): 10.87%						
Model II (HV-containing cycles)							
Intercept	na	na	$\alpha_{0}$	1.998	na	na	na
HV ratio (%)^a^	0% < –≤ 50%	*	$\alpha_{1}$	0.029	0.003	9.194	25.8
Partly snowy dummy	89 cycles	*	$\alpha_{2}$	0.283	0.054	5.877	16.5
Snowy dummy	62 cycles	*	$\alpha_{3}$	0.560	0.048	9.504	26.7
Time dummy (PM = 0, AM = 1)	57 cycles	*	$\alpha_{4}$	−0.453	0.059	−5.911	16.6
Place dummy (Century = 0, Dugald = 1)	22 cycles	*	$\alpha_{5}$	−0.252	0.077	−3.261	9.1
Lane width (ft)^c^	0 ft -1 ft		$\alpha_{6}$	−0.103	0.054	−1.900	5.3
Number of observations (cycles): 314 Prob(F-stats): 0.000	Adjusted R-squared: 0.418 MAPE: 9.72% RMSPE: 12.56%						
Model III (all cycles)							
Intercept	na	na	$\alpha_{0}$	1.966	na	na	na
HV ratio (%)^a^	0% ≤–≤ 50%	*	$\alpha_{1}$	0.023	0.001	19.443	17.3
Partly snowy dummy	539 cycles	*	$\alpha_{2}$	0.326	0.015	21.210	18.8
Snowy dummy	353 cycles	*	$\alpha_{3}$	0.812	0.019	43.298	38.4
Time dummy (PM = 0, AM = 1)	230 cycles	*	$\alpha_{4}$	−0.362	0.023	−15.874	14.1
Place dummy (Century = 0, Dugald = 1)	390 cycles	*	$\alpha_{5}$	−0.164	0.018	−9.320	8.3
Lane width (ft)^c^	0 ft -1 ft	*	$\alpha_{6}$	−0.048	0.014	−3.501	3.1
Number of observations (cycles): 2,212 Prob(F-stats): 0.000	Adjusted R-squared: 0.554 MAPE: 8.49% RMSPE: 11.19%						

*Note*: SE = standard error; na = not applicable.

a The maximum HV ratio is 50%.

b * indicates significant variables with 95% of significance level (P-values < 0.05).

c Lane width indicates the difference from 12 ft.

- Model I for cycles only with PCs
- Model II for cycles with at least one HV
- Model III for all cycles

To compare the degree of influence among the explanatory variables, the t-value ratio (%) was defined for each variable using the following Equation 5.

(5) $$\begin{array}{c} {\left(t-\mathrm{value}\;\mathrm{ratio}\right)}_{i}=100\cdot \frac{\left|t_{i}\right|}{\sum_{i=1}^{6}\left|t_{i}\right|} \end{array}$$

where

$t_{i}$ = t-value for variable i .

The P-values and F-values for the majority of explanatory variables were less than 0.000 for all the models, confirming the statistical significance of the explanatory variables. Considering t-value ratios in each model, snowy condition was the most influential factor in increasing the saturation headway among all explanatory variables, following by partly snowy condition (in Model I and Model III), indicating that deteriorating adverse RW condition is a determining factor for saturation headway. It can also be seen that a 1% increase in the HV ratio increases the saturation headway by more than 0.023 s (Model III) and 0.029 s (Model II). Comparing Model I and Model II, the HV ratio has a 25.9% weight of the total influence. Alternatively, the degree of influence of snowy and partly snowy condition has decreased. This alludes to the influence of the mixture of HVs and the influence of RW condition being comparable.

In addition to these observations, when the “daytime” dummy (defined as the time from sunrise to sunset) was used as an explanatory variable, the P-value was 0.229, that is, rejecting its significance. On the other hand, the “time” dummy had a significant effect, suggesting that driving behavior at AM was more aggressive than PM, and drivers tended to hurry during morning peak regardless of the brightness of the daylight. The negative sign of model coefficient for lane width reveals that expanding the lane width can decrease saturation headways. However, it should be noted that only the range from 12 ft to 13 ft is applicable.

**Figure 8.**
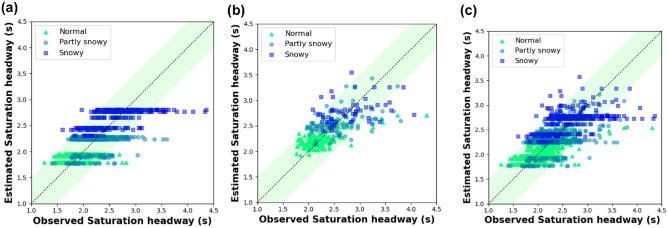
Comparison between observations and estimations: (*a*) Model I, (*b*) Model II, and (*c*) Model III.

Overall, the adjusted coefficient of determination in Model III, that is, 0.544, indicates the significant relationship between independent and dependent variables. The mean absolute percentage error (MAPE) calculated by Equation 6 is 8.49% in the Model III. Figure 8 illustrates the comparison between the observed and estimated saturation headway values. Besides, according to the root mean squared percentage error (RMSPE) of 11.9% provided by Equation 7, this proposed regression model could be further improved. Especially, the proposed model tends to underestimate the saturation headways as the saturation headway per cycle becomes large, and the deviation also becomes more significant under adverse weather conditions. These errors may be because of inter-relationships between various factors triggered by individual variations in driving behavior under adverse RW conditions.

(6) $$\begin{array}{c} \mathrm{MAPE}=\left(\frac{100}{N}\sum_{i=1}^{N}\frac{\left|x_{i}-\hat{x_{i}}\right|}{x_{i}}\right) \end{array}$$

where

$x_{i}$ = observed saturation headways (s), and

$\hat{x_{i}}$ = estimated saturation headways (s).

(7) $$\begin{array}{c} \mathrm{RMSPE}=\left(\sqrt{\frac{100}{N}\sum_{i=1}^{N}{\left(x_{i}-\hat{x_{i}}\right)}^{2}}\right) \end{array}$$

where

$x_{i}$ = observed saturation headways (s), and

$\hat{x_{i}}$ = estimated saturation headways (s).

## Conclusions

In this paper, variations of saturation headway at signalized intersections was investigated under different adverse RW conditions and HV ratios. In addition, regression models were developed to describe saturation headway as function of adverse RW conditions and HV ratios. Road surface condition was the dominant factor, causing from 13.6% to 38% longer headways in partly snow and snowy conditions. Under normal conditions (i.e., either dry, partly wet, or wet), the average saturation headway on the lane adjacent to the median island was 5% shorter than that of the center lane. However, the difference in saturation headway across the lanes became less significant in adverse RW conditions, which could be attributed to a blurred boundary between the lanes as a result of snow accumulation and reduced overtaking through the median lane. Saturation headway distributions were found to follow left-skewed lognormal functions with different parameters, demonstrating increased variance as RW conditions worsened.

In addition, a comparison of the headway among vehicle types showed that HVs required an average of 84% longer headway than PCs. Conversely, the measured PCE under normal conditions was 2.07, while it was 1.72 for partly snowy and 1.47 for snowy, which indicates that HVs are less sensitive to road conditions than PCs. The multiple regression model estimated in this study, with statistically significant variables and a MAPE of less than 10%, is able to estimate the saturation headway according to the HV ratio under different RW conditions. The presence of HVs in cycles itself is found to significantly increase the saturation headway of each cycle; consequently, the agglomerated effects with RW conditions clearly imply that they have notable implications of reducing the performance of signalized intersections. The findings of this study are expected to be helpful for the review of traffic volume and the estimation of SFR in signalized intersections where HVs often travel in severe snowfall conditions.

In closing, as with any research effort, this study has its limitations and the findings open the window for many research extensions that warrant attention. For instance, this paper has used data from two signalized intersections in Winnipeg, Manitoba, and large-scale data collection efforts covering multiple regions will be required to test the geographical stability of saturation headway distributions and models. On the other hand, there is also a possibility of human-oriented errors in the data collection, since pavement conditions and headway data are extracted from the video recordings manually. The future scope of this study is to explore non-linear model specifications to more accurately represent and predict saturation headways. Furthermore, it is also imperative to explore how the saturation headways can be converted to traffic capacity based on the results of this study and to examine the extent to which the study findings are transferable to other geographic locations. The long-term outcomes of this research will help to produce a more generalized signal plan that adapts to the different road weather conditions and HV ratio, and in turn help to improve the traffic capacity and delay conditions at signalized intersections.
